# Ischemia-modified albumin: Crosstalk between fatty acid and cobalt binding

**DOI:** 10.1016/j.plefa.2018.07.014

**Published:** 2018-08

**Authors:** James P.C. Coverdale, Kondwani G.H. Katundu, Amélie I.S. Sobczak, Swati Arya, Claudia A. Blindauer, Alan J. Stewart

**Affiliations:** a Department of Chemistry, University of Warwick, Coventry, United Kingdom; b School of Medicine, University of St Andrews, St Andrews, United Kingdom; c College of Medicine, University of Malawi, Blantyre, Malawi

**Keywords:** Albumin cobalt binding assay, Molecular diagnostics, Free fatty acids, Human serum albumin, Myocardial ischemia, ACB, albumin cobalt-binding, ACS, acute coronary syndromes, ATCUN, amino terminal Cu(II) and Ni(II) binding motif, DTT, dithiothreitol, EPR, electron paramagnetic resonance, EXAFS, extended X-ray absorption fine structure spectroscopy, FFAs, free fatty acids, HRG, histidine-rich glycoprotein, IMA, ischemia-modified albumin, ITC, isothermal titration calorimetry, NMR, nuclear magnetic resonance, NTS, N-terminal binding site on albumin

## Abstract

Myocardial ischemia is difficult to diagnose effectively with still few well-defined biochemical markers for identification in advance, or in the absence of myocardial necrosis. “Ischemia-modified albumin” (IMA), a form of albumin displaying reduced cobalt-binding affinity, is significantly elevated in ischemic patients, and the albumin cobalt-binding (ACB) assay can measure its level indirectly. Elucidating the molecular mechanism underlying the identity of IMA and the ACB assay hinges on understanding metal-binding properties of albumin. Albumin binds most metal ions and harbours four primary metal binding sites: site A, site B, the N-terminal site (NTS), and the free thiol at Cys34. Previous efforts to clarify the identity of IMA and the causes for its reduced cobalt-binding capacity were focused on the NTS site, but the degree of N-terminal modification could not be correlated to the presence of ischemia. More recent work suggested that Co^2+^ ions as used in the ACB assay bind preferentially to site B, then to site A, and finally to the NTS. This insight paved the way for a new consistent molecular basis of the ACB assay: albumin is also the main plasma carrier for free fatty acids (FFAs), and binding of a fatty acid to the high-affinity site FA2 results in conformational changes in albumin which prevent metal binding at site A and partially at site B. Thus, this review advances the hypothesis that high IMA levels in myocardial ischemia and many other conditions originate from high plasma FFA levels hampering the binding of Co^2+^ to sites A and/or B. This is supported by biophysical studies and the co-association of a range of pathological conditions with positive ACB assays and high plasma FFA levels.

## Highlights

• A molecular mechanism underlying the albumin-cobalt binding assay.
• Free fatty acid binding interferes with Co(II) binding to albumin.
• Ischemia-modified albumin corresponds to albumin with increased FFA bound.
• Increased FFA levels are sufficient to explain high ACB readings.
• Clinical data are consistent with FFA as the trigger for high ACB/high IMA levels.

## 1 Introduction

Myocardial ischemia occurs due to restricted blood supply to the muscular tissue of the heart (myocardium) resulting in insufficient oxygen supply. The main cause of this can be the partial or complete blockage of a coronary artery, and a critical depletion of myocardial oxygen leads to cell death, or infarction. Diagnosis of myocardial ischemia typically includes exercise-electrocardiography stress tests, coronary angiography, and imaging stress-echo tests [1]. While a plethora of cardiac biomarkers have been described for detecting the development of other acute coronary syndromes (ACS) [2], [3], there are still few well-defined biochemical markers for identification of myocardial ischemia in advance, or in the absence of myocardial necrosis. One of these biomarkers is based on albumin, the most abundant protein in blood plasma. So-called “ischemia-modified albumin” (IMA) is found to be significantly elevated in ischemic patients [2], [4], [5], [6], [7], and serves as a biomarker for early detection of myocardial ischemia before the onset of irreversible cardiac injury [6]. IMA is solely characterised by its reduced cobalt-binding affinity, which can be measured indirectly by the Food and Drug Administration-approved albumin cobalt-binding (ACB) assay [8], [9].

In the commercially available ACB test, cobalt(II) chloride (approximately 1.5 mol equivalents per albumin molecule) is added to a serum sample, to allow albumin-cobalt binding. Dithiothreitol (DTT), a metal chelator that forms a coloured complex with Co^2+^, is then added. The resulting ill-defined brown DTT-Co^2+^ product is measured by absorption spectrophotometry at 470 nm and compared to a serum-cobalt blank without DTT present. The reduced cobalt-binding capacity of IMA leaves more unbound Co^2+^ to complex with DTT, resulting in higher absorbance readings [10]. The ACB test has an excellent negative predictive value, *i.e.* low IMA readings correspond well to the absence of myocardial ischemia. However, a severe shortcoming is the high incidence of false positives, *i.e.* high readings in the absence of ischemia.

After its first description [8], the molecular identity of IMA remained elusive. Based on the general assumption that Co^2+^ would preferentially bind to an N-terminal site [11], [12], [13], efforts to elucidate the molecular causes of reduced cobalt binding concentrated on this site. It was hypothesized that ischemia causes the N-terminal end of the albumin protein to undergo structural modifications, hence that IMA corresponded to N-terminally modified albumin [13]. The structural modifications proposed and investigated included cleavage of the first two residues and oxidation [11], which were suggested to result from free radical damage, exposure to free iron and copper, or disruption of ion pumps [8], [14].

However, in-depth studies could not reveal a correlation between N-terminal modifications and ACB readings [13], [15]; more recently, no correlation was found between the ACB assay and an enzyme-linked immunosorbent assay that specifically detects N-terminal modification of albumin in patients with either acute coronary syndrome or non-ischemic chest pain [16]. Similarly, patients suffering from acute-on-chronic liver failure have significantly elevated ACB assay readings, but the same proportion of N-terminally modified albumin as healthy individuals [17], [18]. In the light of such findings, low plasma pH as a result of acidosis, and altered plasma cysteine/cystine ratio as a consequence of hypoxia or oxidative stress have also been suspected as molecular causes of reduced cobalt binding [19]. The need to consider the contribution of other plasma components to the Co-DTT complex formation was also highlighted [19]. Indeed, a positive correlation has been identified between the highly elevated serum levels of free fatty acids (FFAs) in patients with acute ischemic myocardia and high levels of IMA [20]. Following our discovery of FFA-mediated inhibition of zinc binding to albumin [21], [22], [23], [24], we have demonstrated that the conformational changes that FFA-binding to albumin elicits in the protein is sufficient to cause reduced cobalt binding capacity [22], [25]. This review will present essential background information on metal ion-albumin interactions and discuss the molecular basis of FFA-mediated inhibition of metal (in particular Co^2+^) binding. It will also provide a clinical perspective to highlight how conclusions from biochemical/bioinorganic investigations are reflected in patient data.

## 2 Albumin – a carrier of essential and xenobiotic metal ions in plasma

Albumin is a ∼66 kDa protein containing 585 amino acids, contributing to around 50% of the total protein concentration in blood plasma, and up to 75% of the colloidal activity [26]. Albumin comprises three homologous but structurally distinct domains, each divided into two sub-domains [27]. One of its key roles in the body is to transport a variety of small molecules, including cholesterol [28], fatty acids [29], and pharmaceutical drugs [30]. Importantly, albumin also serves as an important carrier of inorganic ions, including those required for regular physiological function (Ca^2+^, Cu^2+^_,_ Zn^2+^) [31], toxic metal ions (Cd^2+^ and Ni^2+^) [32], [33], as well as metal-based therapeutics (Au^+^ and Pt^2+^) [34], [35]. Before considering cobalt binding in depth, we will briefly summarise the interactions of albumin with other d-block metal ions, with the exception of Cr^3+^, Fe^3+^, and Mn^2+^, which are preferentially transported by transferrin, another important metal ion transporter in blood plasma. Whilst Fe^3+^ can, in principle, also bind to albumin, this only occurs in cases of severe iron overload [34].

### 2.1 Metal binding sites in serum albumins

**Fig. 1:**
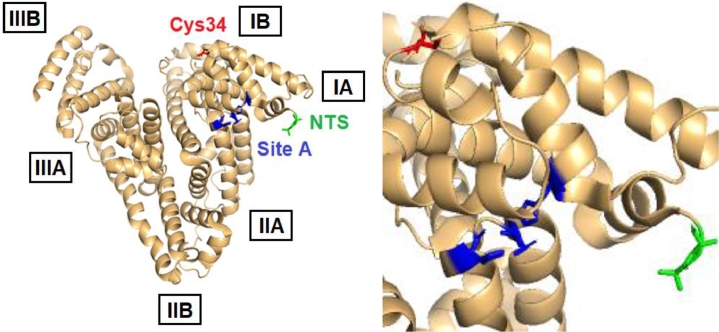
Location of the three metal binding sites that have been successfully identified on human serum albumin,PDB: 5IJF[60]. Site A, the multi-metal binding site (MBS) (blue); NTS/ATCUN motif (green); Cys34 (red). The precise location of site B is not yet known. The boxed labels indicate the six sub-domains of albumin. (For interpretation of the references to colour in this figure legend, the reader is referred to the web version of this article.)

Though originally albumin was thought to transport ions in a non-specific ‘sponge-like’ manner [30], four partially selective metal binding sites have been identified, namely the N-terminal site (NTS), sites A and B, and Cys34 (Fig. 1) [34]. Metal binding to such sites can be studied using a variety of techniques. Stability constants for the binding of d-block metals, including Zn^2+^, Cu^2+^, Ni^2+^ and Cd^2+^, were originally derived from equilibrium dialysis experiments [36], [37], [38], [39]; more recently, isothermal titration calorimetry (ITC) has provided valuable thermodynamic data for metal ion binding [40]. Nevertheless, both of these techniques only provide global binding constants [34] and need to be complemented by techniques that address structural features. For true transition metal ions such as Cu^2+^ and Co^2+^, electronic spectroscopic methods such as circular dichroism allow metal binding to albumin to be studied via transfer of chirality from metal-binding amino acid residues to the d-d/charge-transfer bands of complexed metal ions, providing insight into the geometry of metal-protein interactions [41], [42]. The same ions have unpaired electrons, and can also be investigated using electron paramagnetic resonance (EPR) spectroscopy, which provides insight into the chemical environment surrounding the metal ion [43], [44]. To obtain structural information on the binding of diamagnetic d^10^ ions, such as Zn^2+^ and Cd^2+^, that are largely silent in the aforementioned spectroscopies, nuclear magnetic resonance (NMR) methods have been employed, making use of either partially-characterised ^1^H-resonances of metal-binding residues, or NMR-active nuclei such as the ^111^Cd or ^113^Cd isotopes of cadmium [39], [45], [46], [47]. Further information on the coordination mode, geometry and identification of likely donor ligands has been gained using extended X-ray absorption fine structure spectroscopy (EXAFS) [47]. In addition, mass spectrometry has been used as a tool to detect crosslinking of His67 and His247 by platinum in site A [48].

#### 2.1.1 The N-terminal binding site (NTS)

**Fig. 2:**
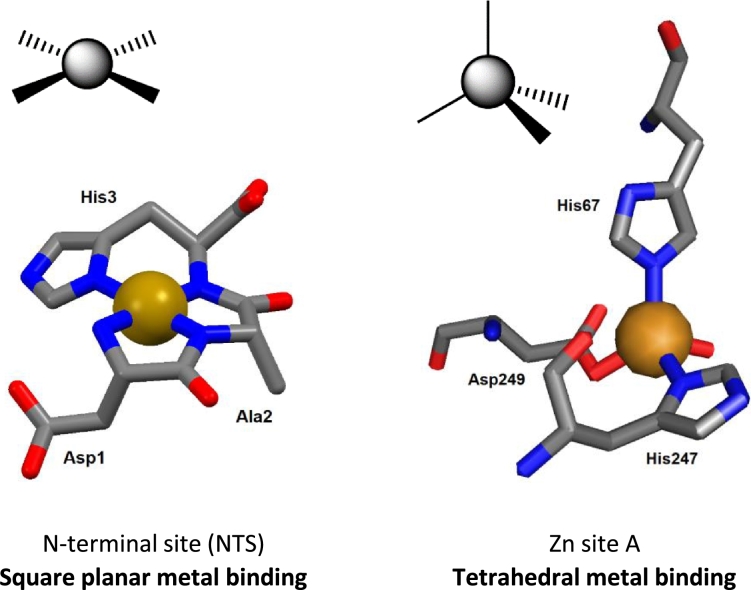
Contrasting geometries of metal binding sites on albumin. Left: square planar coordination of Cu^2+^ or Ni^2+^ at the NTS site; the structure shown is derived from molecular modelling. The N-terminal amino group, two deprotonated backbone amide N atoms and the N(delta) of the imidazole ring of His3 form a square plane around the central metal ion. Right: tetrahedral coordination of Zn^2+^ at site A in human serum albumin (pdb 5ijf). His67 uses its N(epsilon) N atom, whilst His247 binds via N(delta). Asp249 binds in mono-dentate fashion, with the second carboxylate O at *ca.* 2.6 Å distance, too long for a metal-ligand bond. Typically for zinc sites in proteins, angles between ligands deviate substantially from the ideal tetrahedral angle (109.5°) and vary between 95° and 125°. Metal ions are rendered in gold, N atoms in blue, O atoms in red, carbon atoms in grey. No H atoms are shown.

One of the first metal binding sites to be identified on albumin was the N-terminal binding site (NTS), which arises from the first triplet amino acid motif of human albumin: Asp1–Ala2–His3 (Figs. 1 and 2) [49]. It involves the N-terminal amino group, the N(delta) of His3, and two deprotonated backbone amide nitrogen atoms. This square planar configuration of N-donor atoms (Fig. 2) is particularly suitable for Cu^2+^ and Ni^2+^, which has led to the NTS being referred to by the acronym ‘ATCUN’, for the Amino Terminal Cu(II) and Ni(II) binding motif [42], [50]. The ATCUN motif is present in the majority of albumins from different mammalian species, though porcine and canine albumins are notable exceptions, as they lack His3 [34]. Oligopeptide models of the native ATCUN motif have been investigated extensively [34]. The NTS motif is thought to have high conformational flexibility in the absence of bound metal, reflected in the crystal structures of albumin, all of which lack defined structures of the first few N-terminal residues [12]. Interestingly, the N-terminal X-X-His motif is not unique to albumin – many other proteins, such as the peptide hormone Hepcidin, can also bind Ni^2+^ and Cu^2+^ ions via an ATCUN motif [51].

Cu^2+^ binds preferentially to the NTS in albumin, occupying approximately 1–2% of the available NTS – equating to around 15% of total copper in blood plasma [34], [52]. Owing to the d^9^ electronic configuration of Cu^2+^, preference to form square planar complexes, and high stability in the Irving-Williams series, Cu^2+^ is coordinated at the NTS with 1 pM affinity [52], and binds preferentially over other metal ions [22]. Cu^2+^ can also bind at other metal binding sites with comparable or even higher affinities to those of Ni^2+^ and Zn^2+^ [41], however its low relative concentration (10–20 µM total Cu^2+^, and sub-micromolar ‘free’ Cu^2+^ in plasma) [53] compared to albumin means that, in practice, only the NTS is ever occupied by Cu^2+^ [52]. Like Cu^2+^, Ni^2+^ binds to albumin preferentially at the NTS site [33], with micromolar affinity [34]. Ni^2+^ is only present at nanomolar concentrations in plasma, however levels may be elevated under certain pathological conditions (*e.g.* stroke) [54]. Nearly all of plasma Ni^2+^ is albumin-bound [12], [34]. Binding of Ni^2+^ and Cu^2+^ can be modulated by the redox state of Cys34 [43] with higher metal affinity in the reduced (free thiol) state.

#### Site A – the multi-metal binding site

Metal binding site A is located at the interface of domains I and II [34] (Fig. 1), and has been identified and characterised using ^1^H and ^111/113^Cd NMR spectroscopy [39], [45], [46], [55], [56], circular dichroism, site-directed mutagenesis [56], EXAFS [47] and recently X-ray crystallography [57]. As well as having a high nanomolar affinity [23], [24], [38], [39], [47] for the d^10^ divalent cations Zn^2+^ and Cd^2+^, site A can also bind Cu^2+^, Ni^2+^ and Co^2+^ – hence it is also referred to as the ‘multi-metal’ binding site [34], [41], [56]. In fact, up to 90% of the total zinc present in plasma (11.5–36.7 µM total Zn^2+^ in adults [58]) is bound to albumin [59], [60]; this amounts to *ca.* 98% of exchangeable plasma zinc.

EXAFS, site-directed mutagenesis and molecular modelling initially suggested that site A is formed by His67, His247, Asp249, and Asn99 [47], and so distorted trigonal bipyramidal coordination of Zn^2+^ was proposed, with water (or chloride) as the fifth ligand completing the inner coordination sphere. However, the recent X-ray crystallographic structures of human and equine albumins discounted participation of Asn99 and showed site A to be essentially tetrahedral (Fig. 2), with the fourth ligand being a water molecule [57]. Cu^2+^ coordination at site A had also been suggested to be tetrahedral in geometry, as determined by EPR and CD experiments [61]. The combination of amino acid residues bearing intermediate-to-hard N/O-donors [60] (HSAB principle) provide a good coordination environment for metal ions with a small ionic radius and moderate charge (*e.g.* 2+ cations). Notably though, the affinity of site A for Cu^2+^ is 4 orders of magnitude lower than that of the NTS [34], thus site A only becomes populated by Cu^2+^ when more than one molar equivalent of Cu^2+^ is present. Finally, the comparison of apo- and Zn-bound crystal structures of albumin has revealed high structural similarity at site A. Thus, in marked contrast to the flexible NTS, site A is essentially ‘pre-formed’ for metal binding [57], [60]. It is important to note that site A is an inter-domain site, with His67 from domain I, and His247 and Asp249 from domain II.

#### Site B

**Fig. 3:**
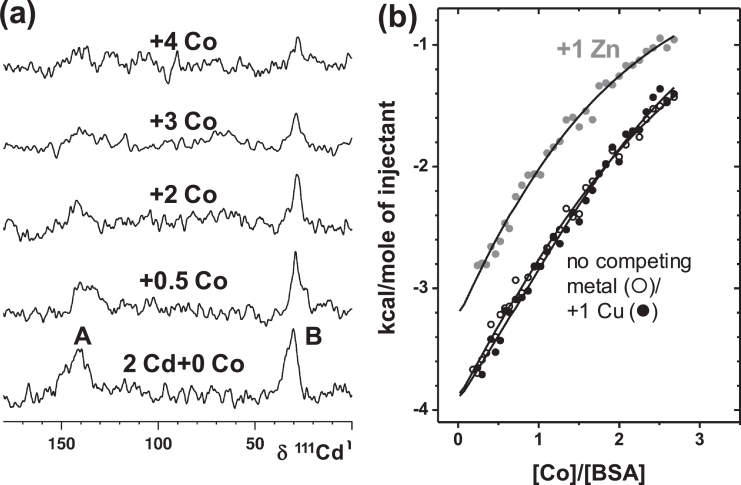
Co^2+^ competes with both Cd^2+^ and Zn^2+^ for albumin binding under physiological conditions (pH 7.4, 50 mM Tris-Cl, 50 mM NaCl) but not with Cu^2+^. (a) 111 Cd NMR spectra of Cd_2_BSA (1.5 mM) with increasing addition of Co^2+^. 111-Cd resonances corresponding to both site A and B (∼140 ppm and 35 ppm, respectively) are affected by Co^2+^. (b) Isothermal calorimetry experiments performed in the presence of 1 mol. equiv. of Cu^2+^ (●) or Zn^2+^ () demonstrate that addition of Zn^2+^ decreases albumin's affinity and capacity for Co^2+^-binding, while addition of Cu^2+^ has no significant effect.

The other Cd^2+^ binding site (site B), which is distinct from site A and the NTS and readily identifiable using ^111^Cd or ^113^Cd NMR (Fig. 3a), appears to bind Cd^2+^ with similar affinity to the multi-metal binding site A [45], [46]. In contrast, site B's affinity for zinc is markedly less than that of site A. Based on NMR data, it is likely that only one nitrogen donor ligand is involved at site B, suggesting this site to be harder (HSAB principle) than site A. The location of site B has remained elusive, but site-directed mutagenesis of His39Leu excluded His39 from involvement in either site A or B [47].

#### Cysteine-34

Albumin contains 17 disulfide bonds, which contribute to the structural stability of the protein. One free thiol residue (Cys34) is located between helices 2 and 3 of subdomain IA (Fig. 1) [34]. Cys34 is not involved in any intramolecular bridging, however it often forms intermolecular disulfides with small sulfur-containing molecules such as cysteine and glutathione [34]. Under normal physiological conditions, approximately 40% of albumin contains ‘reduced’ Cys34 (free thiol) [34]. The restricted location of Cys34 in a crevice of albumin helps to improve its specificity for binding metal ions that favour linear coordination, including Hg^2+^, Au^+^, Ag^+^ and Pt^2+^[34], [35], but not Cd^2+^ or Zn^2+^ [56].

#### Calcium binding sites

Albumin is an important transporter of Ca^2+^ in blood plasma. Many reports suggest that this occurs in a non-specific fashion, involving various carboxylate side chains on the surface of albumin [42], [62], while work by Majorek et al. detected three defined Ca^2+^ binding sites on bovine albumin [63]. It may be significant that one of the Ca^2+^ sites detected by crystallography involves the key site A ligand, Asp248 (corresponding to Asp249 in human albumin) and indeed, Ca^2+^ ions were found to interfere with the ^113^Cd signals for both sites A and B of human albumin [46]. However, the affinity of albumin for Ca^2+^ binding is relatively weak (K_d_ of 0.67 mM), with only around 45% of the 2.4 mM of circulating Ca^2+^ bound to albumin [63], [64]. Mg^2+^, which is also carried by albumin, is thought to bind to the same binding sites as Ca^2+^ but with an even lower affinity (K_d_ of 10 mM) [65].

### 2.2 Cobalt binding to albumin

Cobalt circulates in the blood as Co^2+^ and albumin is its principal transporter in plasma [34]. While it is widely assumed that its binding resembles that of Ni^2+^ and Cu^2+^ (d^8^ and d^9^ metal ions, respectively, with preference for the formation of square planar/tetragonal complexes), Co^2+^ (d^7^) behaves in fact more like Zn^2+^ (d^10^), has a similar ionic radius (0.58 vs. 0.60 Å for Zn^2+^ [66]), and shares a preference for tetrahedral, penta-coordinate or octahedral geometry [67]. For precisely this reason, Co^2+^ has been used extensively as a spectroscopic probe for Zn^2+^in proteins [68], [69].

In total, three significant Co^2+^ binding sites have been identified on albumins – the NTS, site A and site B [42]. Based on Co^2+^ perturbing ^1^H NMR resonances for the three N-terminal residues [11] and Co^2+^ binding to an ATCUN peptide mimic [13], [49], it was assumed that the primary cobalt-binding site was the NTS motif [11], [19], [70]. More recent comprehensive studies on human albumin by Mothes and Faller [71] and Sokolowska et al. [42], and on bovine albumin by our labs [25] have since rejected this claim. Competition with Cd^2+^ and Cu^2+^ monitored by electronic absorption spectroscopy strongly suggested that sites A and B are the preferred Co^2+^ binding sites [16], [42], [71]. Subsequently, ITC and spectroscopic studies identified site B as the strongest cobalt binding site [42]. Co^2+^ binding to sites A and B was also confirmed by ^111^Cd NMR spectroscopy for bovine albumin (Fig. 3a), and competition with Zn^2+^ was evident from ITC (Fig. 3b) [25]. In contrast, blocking the NTS site with Cu^2+^ did not impart any significant effect on Co^2+^ binding [25], [71]. It is important to note however, that even though Co^2+^ and Zn^2+^may be regarded as metal ions with similar properties, the apparent binding constant for Co^2+^ binding to its strongest site on bovine albumin (log *K*_app_ = 4.6 ± 0.3 × 10^4^ M^−1^; Fig. 5b; [25]) and 9 ± 5 × 10^4^ M^−1^ for human albumin [42]) were around one order of magnitude lower than those determined for Zn^2+^ [25].

In summary, even though all three apparent binding constants for Co^2+^ binding to human albumin lie between 9 ± 5 × 10^4^ M^−1^ and 0.9 ± 0.3 × 10^4^ M^−1^ [42], and hence the respective equilibria do overlap, the NTS site is now known to have the weakest affinity for Co^2+^ [42]. Most importantly, this weaker than anticipated binding of Co^2+^ to the NTS means that the initially proposed molecular basis of the ACB assay to assess the likelihood of myocardial infarction required revision, since the original studies assumed that Co^2+^ binds exclusively to the NTS [8], [19].

## 3 Free fatty acid binding to albumin and allosteric inhibition of metal ion binding

Albumin has an unparalleled capacity to bind and transports a range of organic molecules under physiological conditions [72]. Notable among those transported are FFAs, important substrates in organismal metabolism for which albumin is the main carrier [73], [74], [75], [76]. FFAs are the main source of energy for heart and skeletal muscle. Disturbances of the levels and/or distribution of fatty acids in the body have been linked to a spectrum of pathological disorders, including diabetes, cardiovascular and neurological diseases, and cancer [77]. Owing to the abundance of albumin in plasma, and the importance of fatty acids in metabolism and disease progression, binding of FFAs to albumin has been studied intensively in the past four decades [73], in particular by X-ray crystallography [75], [78], [79] and ^13^C NMR spectroscopy [80], [81].

**Fig. 4:**
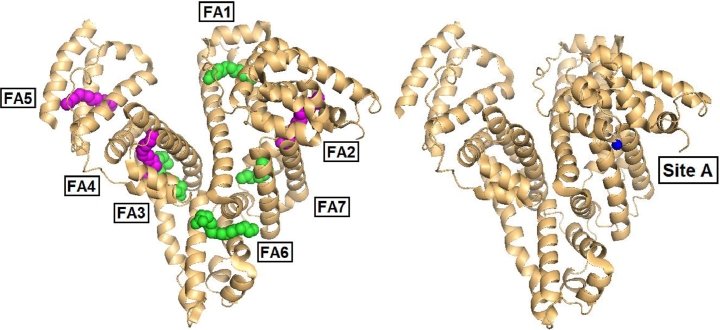
(a) Location of fatty acid (FFA) binding sites FA1-7 on human serum albumin (PDB: 1E7H), complexed with hexadecanoic (palmitic) acid [82]. High (magenta) and low (green) affinity sites are shown. (b) Location of site A, the multi-metal binding site (PDB: 5IJF), occupied by Zn^2+^ (blue) [57]. Site A and FA2 are both located between subdomains IA-IIA. The inter-domain nature and the proximity of FA2 to site A allows for the allosteric switching of metal ion binding [21].

Up to seven medium-to-long chain (C10-C18) fatty acid binding sites (FA1-7) have been identified on albumin, spread over the three domains (see Table 1 and Fig. 4) [75]. The binding affinities depend on both the site and the FFA chain length. Four additional binding locations have been described for short-to-medium chain fatty acids [82], however for the purposes of this review we will focus on FA1-7 (Fig. 4). In a normal physiological state, albumin circulates with between 0.1–2 equivalents of FFAs bound, however it pathologically can bind in excess of 6 equivalents [83]. The seven identified binding sites can be broadly split into two categories: the high-affinity sites (FA2, FA4 and FA5) and the low-affinity sites (FA1, FA3, FA6 and FA7) [83]. The high-affinity site FA2 is close to metal-binding site A and therefore of particular interest. This relatively hydrophobic site is, like metal site A, an inter-domain site and is located between sub-domains IA and IIA (Fig. 4) [82]. Compared to FFA-free albumin, accommodation of a fatty acid molecule in site FA2 requires a change in the mutual arrangement of domains I and II. While short-chain FFAs (<C8) were originally thought to be too short to successfully dock in the FA2 site [82], more recent ^1^H and ^111^Cd NMR studies indicated that octanoate can bind to this site. Molecular modelling suggested that the half-pocket in domain II is sufficient to accommodate octanoate, and therefore does not require the domain-domain movement [25].

**Table 1:** The location and characteristics of fatty acid binding sites FA1-7 of albumin. Particular attention is drawn to binding site FA2, since occupation of this site by FFAs causes an allosteric switch in metal binding at site A, owing to its close proximity; both are located between subdomains IA and IIA.

Site	Affinity	Physiological^a^	Subdomain	Comments	Reference
FA1	Low	–	IB	Site is relatively accessible to solvent	[79], [82]
FA2	High	Yes	IA-IIA	Allosteric switch affecting site A	[21], [82], [83]
FA3	Low	–	IIB-IIIA	Chain distorted in longer FFAs	[78], [82]
FA4	High	Yes	IIIA	Inverted configuration for C18 FFAs	[82], [83], [143]
FA5	High	Yes	IIIB	C18 FFAs accommodated	[82], [83]
FA6	Low	–	IIA-IIB	Absence of ligands for carboxylate	[78], [79], [82]
FA7	Low	–	IIA	Preference for shorter-chain FFAs	[78], [82]

a (Partially) Occupied under basal physiological conditions (pH 7.4, 0.5–2 mol. equiv. of FFA).

**Fig. 5:**
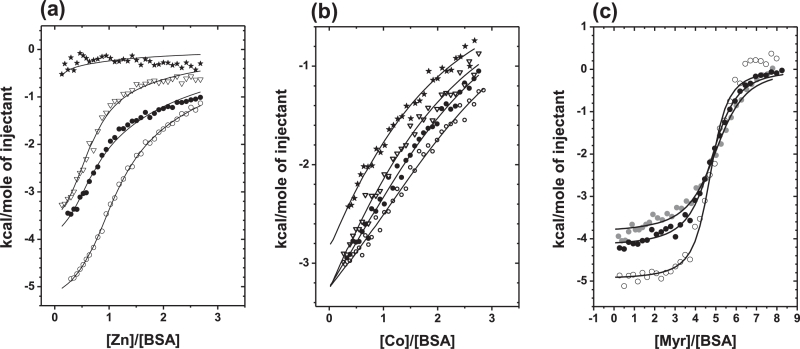
Isothermal titration calorimetry experiments demonstrate the mutual modulation of metal and fatty acid binding to bovine albumin. The presence of the C14:0 fatty acid myristate (○, 0 mol. equiv.; ●, 1 mol. equiv.; ▽, 3 mol. equiv.; and ★ 5 mol. equiv.) affects the binding capacity of albumin for Zn^2+^ (a) and Co^2+^ (b) under near-physiological conditions (pH 7.4, 50 mM Tris-Cl, 50 mM NaCl). Co^2+^ binding to albumin is not only weaker than that of Zn^2+^, but the effect of FFAs on Zn^2+^ binding is also much more pronounced than that of Co^2+^. (c) The presence of 1 mol. equiv. of Zn^2+^ () or Co^2+^ (●) affects the energetics of fatty acid binding relative to the metal-free experiment (○), likely due to the need to remove the metal before the FFA can bind. Notably, again the effect for Zn^2+^ is larger than that for Co^2+^.

While metal site A is essentially ‘pre-formed’ for metal (physiologically Zn^2+^) binding in FFA-free albumin [60], this is not the case when FA2 is occupied by a longer chain FFA (*e.g.* myristic acid, C14), as the distance between the metal-coordinating residues is too large after the conformational change [21], [24], [56]. This crucial discovery suggested that FFA and zinc concentration(s) in blood plasma may be correlated through an allosteric mechanism based on albumin [21], [22]. Competition experiments monitored by ITC demonstrated that the zinc-binding capacity of both bovine and human albumin for site A was dramatically reduced [23], [25]. Five equivalents of myristate were sufficient to completely inhibit Zn^2+^coordination to site A in bovine albumin (Fig. 5a), with site B also affected more or less severely [25]. Importantly, FA2 is one of the high affinity sites, and will become significantly populated already at 1 molar equivalent [83], [84]. Indeed, the data in Fig. 5a indicate that the largest effect is seen between 0 and 2 molar equivalents. The downstream implications of this allosteric switch for the fate of plasma zinc are discussed elsewhere [21], [22], [23].

**Fig. 6:**
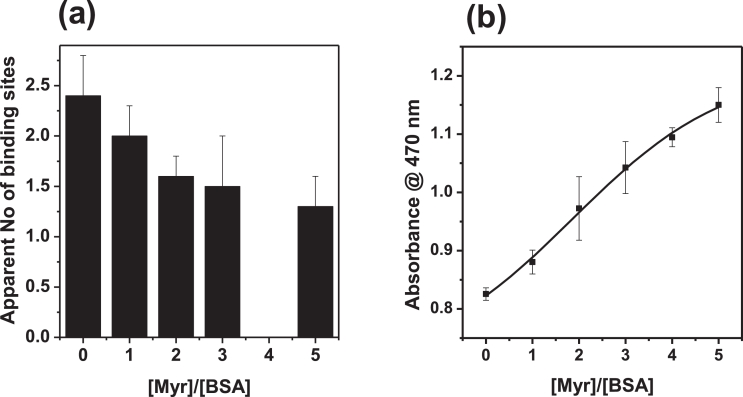
Increasing FFA (myristate, C14:0) decreases the total Co^2+^ binding capacity of BSA, (a) reflected in the number of apparent binding sites of albumin for Co^2+^ (No data for 4 mol. eq. Myr). (b) In turn, this affects the formation of the Co-DTT complex as part of the ACB assay (b), used for the detection of myocardial ischemia.

Crucially, although the binding preferences of Zn^2+^ and Co^2+^ are not identical, the presence of myristate also clearly reduced the binding capacity of bovine albumin for Co^2+^ (Fig. 5b) [25]. The effect on Co^2+^ binding is less severe than that on Zn^2+^ binding, with 5 molar equivalents of myristate reducing binding by *ca.* 50% [25]. This is in agreement with the fact that Co^2+^ does not bind preferentially to site A, but site B (which in BSA is affected by FFA binding, but less severely) [25], and can also bind to the NTS motif which is not expected to be adversely affected by the presence of FFA. Similarly to Zn^2+^, it appears that the bound metal ion must first be removed from site A before fatty acid binding can occur at FA2, identified by a reduction in the exothermicity of the FFA-binding reaction (Fig. 5c) [25]. The number of apparent Co^2+^ binding sites in these experiments was in broad agreement with other experimental data, although we note that the selected experimental conditions did not allow to fully saturate all three binding sites. The apparent number of binding sites in the absence of myristate amounted to 2.4, and reduced to *ca*. 1.3 sites, implying that (at least) one binding site became non-functional (Fig. 6a). This is consistent with an inhibition of cobalt-binding to site A, as a result of FFA binding to the nearby FA2 site [25]. Most importantly, increasing the levels of FFA in a mock ACB assay is sufficient to lead to increased formation of the Co-DTT product, with concomitant higher absorbance readings (Fig. 6b). The magnitude of the changes in absorbance at 470 nm is broadly in line with effects seen in clinical studies [25]. We next explore whether this molecular mechanism may be reflected in clinical data.

## 4 Ischemia-modified albumin in disease states

As indicated previously, the diagnostic specificity of the ACB assay is very low, resulting in a high proportion of false positives, *i.e.* high readings despite the absence of ischemia [15], [85]. This realisation has motivated a large number of studies which found positive ACB readings for a wide range of disease conditions including ACS [20], [86], chronic liver and kidney diseases [87], [88], infectious diseases such as malaria [89], and pregnancy-related conditions such as pre-eclampsia [90]. In addition, elevated IMA levels have been measured in metabolic syndrome [91], [92], diabetes [93] and obesity [94], [95] while exercise and trauma have also been investigated [96], [97]. These conditions, therefore, should have a common feature that can explain elevated IMA levels, and we propose that this common feature is elevated plasma FFAs. The latter have been shown to independently influence the ACB assay to the same extent as ACS and other conditions [20], [25]. Together with the biochemical and biophysical studies detailed in Section 3, it is compelling to suggest that IMA corresponds to albumins in which FA2 is occupied. To further explore this hypothesis, Table 2 compiles selected conditions which are positive for the ACB assay and reports quantitative data on serum FFAs drawn from the literature. Cobalt binding to albumin is both specific and proportional to the total serum albumin concentration, and so many studies adjust for the total albumin level [10].

**Table 2:** Selected conditions associated with a positive ischemia modified albumin (IMA) test and increased free fatty acids (FFAs) with the corresponding IMA and FFA levels.

Condition			IMA levels	Controls	References (IMA levels)	Plasma /serum FFA levels	Controls	References (FFA levels)
ACS	ST-segment elevated myocardial infarction		92.1 (±10.6) Abs units/mL	77.9 (±6.69) Abs units/mL	[86]	840 (±320) µM	750 (±280) µM	[98]
	Non-ST-segment elevated myocardial infarction		87.3 (±5.95) Abs units/mL					
	Unstable angina		88.9 (±6.16) Abs units/mL					
	Acute myocardial infarction		119 (±37.3) Abs units/mL	88.6 (±19.3) Abs units/mL	[20]	1030 (±450) µM	770 (±340) µM	[20]
Acute ischemic stroke			1.180 (±0.223) Abs units	0.820 (±0.129) Abs units	[144]	530 (350–710) µM	240 (120–380) µM	[145]
Obstructive sleep apnea syndrome			0.58 (±0.11) Abs units	0.43 (±0.09) Abs units	[128]	Proposed higher levels, increase with period of oxygen desaturation	no data	[146], [147]
Diabetes		Diabetes only	0.478 (±0.095) Abs units	0.395 (±0.054) Abs units	[114]	>750 µM	<550 µM	[148]
		Diabetic foot	0.721 (±0.123) Abs units					
Rheumatoid arthritis			0.495 (±0.01) Abs units	0.433 (±0.02) Abs units	[117]	(0.59 (0.47–0.65) mM	0.40 (0.35–0.50) mM	[149]
Ankylosing spondylitis			0.44 (±0.17) Abs units	0.32 (±0.13) Abs units	[118]	883.89 (±55.32) µg/mL	760.84 (±31.40) µg/mL	[150]
Psoriasis			0.85 (±0.15) Abs units	0.79 (±0.09) Abs units	[126]	No global increase but increases in C16:1n-7, C18:2n-6, C18:3n-3, C20:0	no data	[151]
Chronic liver disease			0.532 (±0.168) Abs units	0.320 (±0.126) Abs units	[152]	620 (120–3400) µM	450 (110 – 900) µM	[87]
Chronic renal disease			0.357 (±0.083) Abs units	no data	[88]	492.63 (±143.59) µM	302.65 (±142.18) µM	[111]
Subclinical hypothyroidism			no data	0.41 (±0.06) Abs units	[115]	∼675 (±) µM	∼325 (±) µM	[153]
Sepsis			0.967 (±0.734) Abs units	0.007 (±0.009) Abs units	[119]	4 fold increase	no data	[154]
Malaria			0.56 (±0.13) Abs units	0.24 (±0.04) Abs units	[89]	2.17 fold increase	no data	[120]
Trauma			0.63 (±0.18) Abs units	0.39 (±0.05) Abs units	[97]	2010 (±190) µM	no data	[124]
Ovarian torsion			0.704 (±0.059) Abs units	0.667 (±0.052) Abs units	[129]	no data	no data	no data
Polycystic ovarian syndrome			0.52 (0.21–1.12) Abs units	0.35 (0.06–0.90) Abs units	[127]	Total levels unknown but increase in C16:0 and C18:1n9cis	no data	[155]
Mothers bearing small-for-gestational-age foetuses			IMA/albumin: 1.28 (±0.17) g/dLin 1st semester	IMA/albumin: 1.16 (±0.21) g/dL in 1st semester	[116]	no data	no data	no data
Intrauterine growth restriction			78.7 (±6.9) Abs units/mL	74.4 (±7.8) Abs units/mL	[156]	355 µM (in amniotic fluid)	125 µM (in amniotic fluid)	[157]
Preterm babies with respiratory distress syndrome			0.91 (±0.15) Abs units	0.63 (±0.12) Abs units	[130]	no data	no data	no data
Exercise			0.324 (±0.039) Abs units	0.281 (±0.052) Abs units	[96]	>2000 µM	< 600 µM	[158]

ACSs are well-known to be associated with increased serum FFA concentrations [20], [98]. The pain and the stress associated with such syndromes is thought to trigger a sympathetic discharge, with the release of catecholamines which activate hormone-sensitive tissue lipase – the enzyme which hydrolyses triglycerides and hence liberates FFAs into the circulation [99], [100], [101]. This leads to elevated serum free fatty acid concentrations within 1–2 hours from the onset of ACS, and the degree of increase in FFA concentration has been positively associated with serious ventricular arrhythmias [102]. Significantly, the ACB assay values are also positively correlated to the severity of the ACS condition [20], [86], [102]. In addition, the IMA levels detected via the ACB assay increase within minutes of the onset of ischemia, stay high for 6 to 12 hours before returning to normal level within 24 hours. This correlates to similar changes in FFA levels, which return to normal after 24 to 48 hours after myocardial ischemia [103], but is in contrast to explanations invoking N-terminally modified albumin, as albumin has a half-life of *ca.* 20 days and so IMA should be detected for several days following ischemia [104], [105].

Higher FFA concentrations in plasma have been observed in several non-communicable diseases which also result in a positive ACB assay [22]. In fatty liver disease for example, there is insulin resistance which causes a withdrawal of the inhibition of dephosphorylation of hormone-sensitive lipase activity to reduce fat hydrolysis [106], [107], [108]. Further to this, the capacity of the liver to utilise and export FFAs is impaired, leading to increased FFAs in the circulation [87], [109], [110]. Similarly, chronic kidney disease is associated with raised FFA concentrations arising from TNF-*α*-induced adipose tissue lipolysis as a consequence of systemic inflammation [111], [112]. In addition, when patients suffering from metabolic syndrome are given high-fat diets, a significant increase of their IMA/albumin ratio occurs [113]. It is therefore consistent with our hypothesis that those disease states are associated with positive ACB assays. Further conditions with positive ACB readings include diabetes [114], hypothyroidism [115], intrauterine growth restriction [116], chronic inflammation (rheumatoid arthritis [117] and ankylosing spondylitis [118]), infection (sepsis [119] and malaria [89]), exercise [96] and trauma [97]. All of these are also associated with high FFA concentrations (see Table 2) through various physiological and pathophysiological pathways [120], [121], [122], [123], [124], [125].

However, for some other conditions associated with high IMA levels (psoriasis [126] and polycystic ovarian syndrome [127]) no variation in FFA levels compared to healthy controls have been found. Yet some specific long-chain FFAs were measured at higher concentrations and their increased binding affinity for albumin may explain the observed changes in albumin metal-binding capacities [82]. For other conditions (obstructive sleep apnoea syndrome [128], ovarian torsion [129], mothers bearing small-for-gestational-age foetuses [116] and preterm babies with respiratory distress syndrome [130]), FFA levels have not yet been measured. Several other studies detected higher IMA levels in yet more conditions (hyperemesis gravidarum [131], perinatal asphyxia [132], mild cognitive impairment [133], pre-eclampsia [90]), however they were not included in our analysis as “IMA levels” were measured with an immunoassay (see next section) instead of the ACB assay.

## 5 Proposed alternatives to the ACB assay

An enzyme-linked immunosorbent assay has been developed as an alternative to the ACB assay to specifically detect N-terminal modification of albumin. However, no correlation has been found between the results of this assay and IMA levels measured via the ACB assay in patients with either acute coronary syndrome or non-ischemic chest pain [16]. This is consistent with metal binding sites A and B playing a more important role in cobalt binding than the N-terminus.

Other studies on human serum albumin have utilised Cu^2+^ and Ni^2+^ instead of Co^2+^to assess reduced metal binding. In some cases, these studies were inspired by the originally proposed mechanism involving binding to the NTS [134], [135], [136]. Even though Cu^2+^ and Ni^2+^ do indeed preferentially bind to the N-terminus, these studies were successful in demonstrating poor binding capacity of albumin for these ions in coronary artery syndromes – similar to the ACB assay [134], [135], [136]. It should however be considered that site A is a potent secondary binding site for these metal ions once the NTS is saturated, as explained in Section 2 [34], [137], [138]. Therefore, providing that such tests employ an appropriate metal: albumin molar ratio (≥2), FFAs can affect the binding capacity of albumin for Cu^2+^ and Ni^2+^ binding to site A (and site B) like for Zn^2+^ or Co^2+^ [8], [22], [135]. Most recently, a ^13^C NMR-based protocol using ^13^C-methyl-labeled oleic acid (OA) as a reporter molecule has also been developed to measure the amount of long chain FFAs bound to albumin as an alternative to the ACB assay that is not dependent on total albumin concentrations [139].

## 6 Conclusion

Use of the ACB assay to measure IMA levels in multiple pathological conditions has gained traction in recent years. The diagnostic value of this test critically depends on understanding its molecular basis. In the light of compelling evidence, there is now increasing recognition of the fact that N-terminal modification is not a plausible explanation for reduced cobalt binding by albumin [16], [139], [140]. Nonetheless, the FFA-based mechanism is not yet widely accepted either, with many recent studies claiming that IMA corresponds to a marker for “oxidative stress”. In principle, an altered redox balance may well affect the ill-defined chemistry of complex formation between Co^2+^ and DTT, as both agents are prone to oxidation. This alternative hypothesis which does not require covalent modification of albumin may also be more compatible with the timescales of increased and returned to normal ACB readings. At present, corresponding quantitative data and experiments to demonstrate the viability of this hypothesis are scarce, and it leaves unclear the role of albumin in the readout, although the possibility of ternary complex formation was raised [19]. The correlation between ACB assay readings and FFA levels is clear (Fig. 6b), provides a coherent explanation of the chemical identity of IMA, and is consistent with all clinical observations. Serum FFA, in particular unbound FFA, concentrations are useful biomarkers for early diagnosis of ACS [141]. We suggest that the ACB asay – or indeed one of its variants using other metal ions – may be re-purposed as a test for increased serum FFAs [22], [25], [140], [142]. A comprehensive understanding of the chemical species contributing to the overall readouts, including effects of pH and redox chemistry, should enable the design of a test with much better specificity and diagnostic value.

## Conflict of interest

There are no financial or other relationships that might lead to a conflict of interest for the authors.
